# Profiling the Anaerobic Response of *C. elegans* Using GC-MS

**DOI:** 10.1371/journal.pone.0046140

**Published:** 2012-09-27

**Authors:** Jeffrey A. Butler, Robert J. Mishur, Alex F. Bokov, Kevin W. Hakala, Susan T. Weintraub, Shane L. Rea

**Affiliations:** 1 The Barshop Institute for Longevity and Aging Studies, University of Texas Health Science Center at San Antonio, San Antonio, Texas, United States of America; 2 Department of Physiology, University of Texas Health Science Center at San Antonio, San Antonio, Texas, United States of America; 3 Department of Biochemistry, University of Texas Health Science Center at San Antonio, San Antonio, Texas, United States of America; University College Dublin, Ireland

## Abstract

The nematode *Caenorhabditis elegans* is a model organism that has seen extensive use over the last four decades in multiple areas of investigation. In this study we explore the response of the nematode *Caenorhabditis elegans* to acute anoxia using gas-chromatography mass-spectrometry (GC-MS). We focus on the readily-accessible worm exometabolome to show that *C. elegans* are mixed acid fermenters that utilize several metabolic pathways in unconventional ways to remove reducing equivalents – including partial reversal of branched-chain amino acid catabolism and a potentially novel use of the glyoxylate pathway. In doing so, we provide detailed methods for the collection and analysis of excreted metabolites that, with minimal adjustment, should be applicable to many other species. We also describe a procedure for collecting highly volatile compounds from *C. elegans*. We are distributing our mass spectral library in an effort to facilitate wider use of metabolomics.

## Introduction

*Caenorhabditis elegans* is a model organism that has seen heavy exploitation as a research tool over the last four decades. Its use has led to fundamental discoveries in fields such as aging, apoptosis, development, and neurobiology [1], [2], [3], [4]. One unusual feature of this organism, that is not commonly appreciated, is that it is a facultative anaerobe: In the presence of oxygen it makes ATP by aerobic respiration, but during periods of oxygen deprivation it switches to fermentative ATP generation. Wild-type worms can survive up to three days of anoxia - some *daf-2* mutants even considerably longer [5], [6]. Various studies over the years [7], [8] have suggested that worms might utilize several fermentation pathways, one of which has been proposed to exploit a fumarate reductase that allows charging of the inner mitochondrial membrane and continued operation of complex V [9]. Under conditions of oxygen deprivation, we might therefore predict there are multiple alterations to the worm's intermediary metabolism. The global perspective afforded by a metabolomics approach is perfectly suited to investigate the nature and extent of such predicted changes.

In this study, we used gas-chromatography-mass spectrometry (GC-MS) to probe the metabolic differences between wild-type *C. elegans* exposed to normoxia versus anoxia. We focus our efforts on the exometabolome – the ensemble of metabolic end-products excreted into the collection media during the analysis period. Exometabolites are distinguishable from intestinal defecatory waste products in being compounds derived from cellular metabolism. Exometabolomic mapping is equivalent to metabolic footprinting [10], [11], [12], [13], [14], and analysis of this compartment, rather than assessment of the full metabolome, was chosen as a means to reduce sample complexity, and as a way to highlight flux through those pathways of intermediary metabolism that were operative within the cellular ensemble during the collection period.

We find that the *C. elegans* exometabolome consists of only a couple hundred detectable compounds; this compares with the potentially thousands of chemical species present inside cells [15]. We also discover several unappreciated mechanisms by which *C. elegans* survives anoxia, from simultaneous operation of fermentation reactions to partial reversal of branched-chain amino acid catabolic pathways, as well as a potentially novel use for the glyoxylate pathway. The analytical techniques described in the current study should find broad appeal since most laboratories interested in undertaking metabolomics studies will be able to implement them. We are also distributing a library of compiled spectra of known and unknown compounds detected in the exometabolome of *C. elegans* to facilitate further metabolomic studies in this and other species (File S1).

## Results

In previous studies, we established techniques for collecting the exometabolome of large, synchronous populations of *C. elegans* [5]. Analysis of this compartment by HPLC-UV was insufficient to resolve all but a few compounds. Nonetheless, PCA of the resulting multi-compound peaks was able to differentiate aerobically-cultured worms from anaerobically-cultured ones. Here we extend those studies and use GC-MS for metabolite separation and detection.

Synchronous populations of wild-type worms (N2) were cultured to gravid adulthood and subsequently incubated either under atmospheric conditions or in the absence of oxygen for a period of 18 hours. Excreted metabolites were then collected and samples were dried overnight and derivatized with methoxyamine and *tert*-butyldimethylsilane (*t*-BDMS) to increase stability and GC volatility. To observe highly volatile components an alternate procedure was established utilizing ether extraction: this step was applied prior to derivatization and in lieu of the drying step to prevent metabolite loss (Materials & Methods). A modified GC temperature profile was also used with this latter approach in order to prevent volatiles eluting with the solvent front. Propionic acid eluted only slightly later than the solvent front and as a consequence data for just 3 of 5 replicates was obtained. To deconvolute individual mass spectra in GC peaks containing multiple compounds, we used the freely-available *AMDIS* (Automated Mass Spectral Deconvolution and Identification System) software [16]. This program identifies patterns of ions with different mass-to-charge ratios (*m/z*) that have nearly-identical elution profiles. *AMDIS* is especially efficacious when only a small number of compounds co-elute. Since we detected ∼200 compounds across the entire temporal length of our analysis, metabolite co-elution within peaks was manageable.

**Figure 1:**
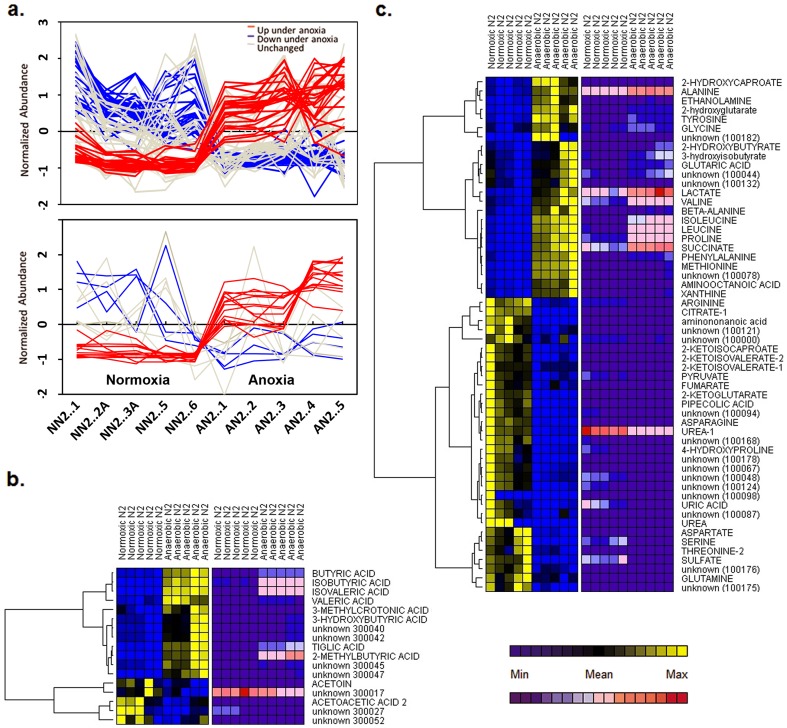
GC-MS reveals marked differences in the exometabolome of *C. elegans* cultured under anaerobic versus normoxic conditions. (**a**) Extracted peak areas for all metabolites were log transformed after normalization to an internal standard and total protein. Metabolites that differed significantly between conditions were identified using a mixed-modeling approach. A false discovery rate (FDR) of 5% was used to set the significance cutoffs. Shown are spaghetti plots for metabolites analyzed by the two different GC procedures described in Methods. Lines correspond to individual metabolites: red lines, significant increases under anoxia; blue lines, significant decreases; and gray lines, no significant change. To aid visual interpretation, the values plotted in this panel were scaled by being converted to z-scores. (**b** and **c**) Hierarchical clustering (based on Pearson's correlation coefficient) was used to segregate metabolites that were synchronously up- or down-regulated following oxygen removal (only significantly altered metabolites are plotted). A low-abundance cut-off filter was applied. Heat maps are colored according to: (i) individual metabolite variation across the sample set (blue-yellow); and (ii) global variation among metabolites over the entire exometabolome data set (blue-red). The latter method only approximates relative metabolite abundance (compare to Figure S1). Exometabolites are plotted in two groups based on analytical separation technique, with volatile metabolites shown in (**b**) and remaining components in (**c**).

**Figure 2:**
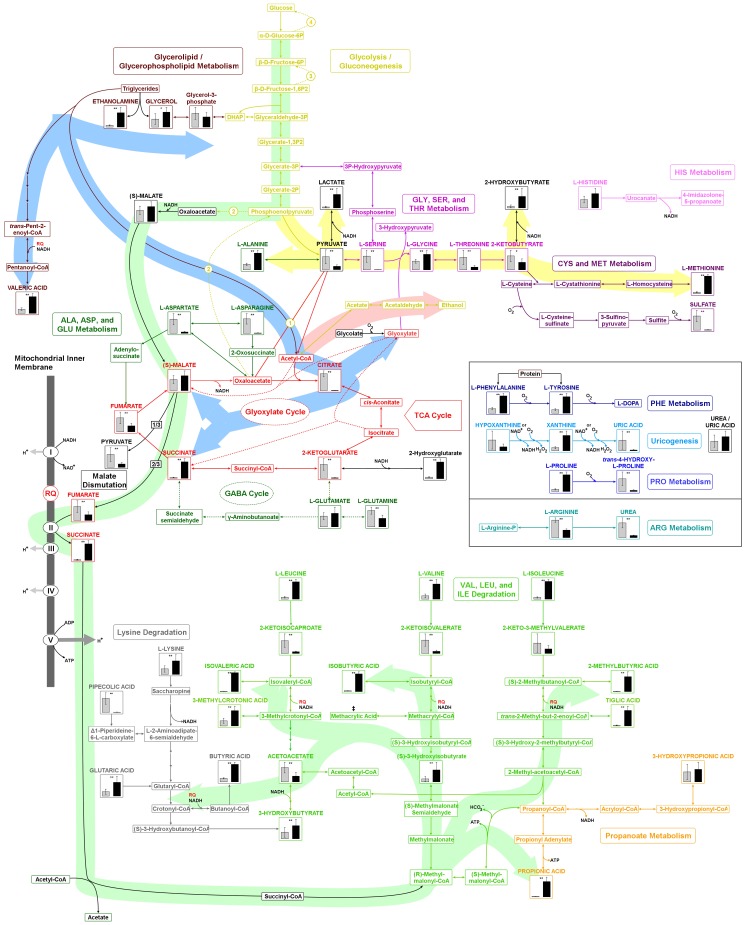
Metabolic map illustrating intracellular changes predicted to occur based on the exometabolome of wild-type worms following exposure to 18 hours of anoxia. Major metabolic alterations characterize the *C. elegans* response to anoxia. Exometabolites that were identified by GC-MS, and which were used to drive map construction, are accompanied by bar graphs. In each graph, the incubation condition that resulted in the highest level of expression of a particular metabolite was assigned an expression value of one then the value in the other condition was scaled accordingly (*grey*: normoxic, *black*: anaerobic). Shown are the averages of five measurements, with error bars representing ± one standard deviation. Major flux pathways are color-coded as follows: *green*, glycolysis/malate dismutation/volatile fatty acid synthesis; *blue*, glyoxylate cycle; *pink*, acetate/ethanol fermentation; *yellow*, lactate fermentation/2-hydroxybutyrate fermentation. *, less than 10% false discovery rate; **, less than 5% false discovery rate; NADH, nicotinamide adenine dinucleotide-producing reactions; RQ, reduced rhodoquinone; O_2_, oxygen consuming reactions; ^‡^, methylacrylic acid was detected but resided on a shoulder of a background peak that we could not deconvolve fully. Circled numbers represent the following glycolysis by-pass enzymes of gluconeogenesis: 1, pyruvate carboxylase; 2, phosphoenolpyruvate carboxykinase; 3, fructose 1,6-bisphosphatase; 4, glucose-6-phosphatase. (Although not shown in the map, pyruvate is the source of acetyl CoA in the coupled conversion of succinate to succinyl CoA on the path to volatile fatty acid synthesis.)

To identify components comprising the *C. elegans* exometabolome, mass spectra were searched against a mass spectral library of *t*-BDMS derivatives (File S2). This library was curated by augmenting a freely-available library with spectra of purified compounds run on our instruments (identified in figures using capital letters). Compounds which matched library entries but which weren't confirmed by running standards are annotated using lower case letters. Artifacts arising from derivatization reagents were identified based on their presence in a blank sample (incubation media only) and are accordingly annotated within the attached library. To quantify specific metabolites, extracted ion chromatograms were integrated using the freely-available program *MET-IDEA (v.2.0)*. All metabolite levels within a sample were first normalized against an internal standard (3,4-dimethoxybenzoic acid), then normalized on the basis of total worm protein. For select metabolites (see Discussion), four-point calibration curves were prepared using standard compounds to allow absolute quantities to be calculated (Figure S1). Metabolites that differed significantly between animals exposed to anaerobic versus normoxic conditions are illustrated in Figure 1. Of the compounds detected using our ‘volatile compounds’ method, several reduced fatty acid components, including butyric acid, isobutyric acid, isovaleric acid, 2-methylbutyric acid, 3-methylcrotonic acid, propionic acid and tiglic acid were found to be significantly elevated in the exometabolome of worms exposed to anaerobic conditions (Figure 1b and File S1). Using our ‘standard method’ we found several amino acids, including alanine, glycine, isoleucine, methionine, proline, leucine and valine, were significantly elevated in anaerobically-cultured worms. In contrast, both arginine and serine were significantly reduced. We also observed a significant decrease in two of the three branched-chain keto acids (2-ketoisocaproic acid and 2-ketoisovaleric acid, Figure 1c). The other branched-chain keto acid, 2-keto-3-methylvaleric acid, was significantly reduced in three of five sample sets. To facilitate biological interpretation of our data, we plotted differentially-regulated metabolites onto a curated map (KEGG [17], [18], [19]) of *C. elegans* intermediary metabolism (Figure 2). NemaPath [20] was used to fill in obvious enzyme gaps by using a slightly more relaxed orthology threshold filter.

**Figure 3:**
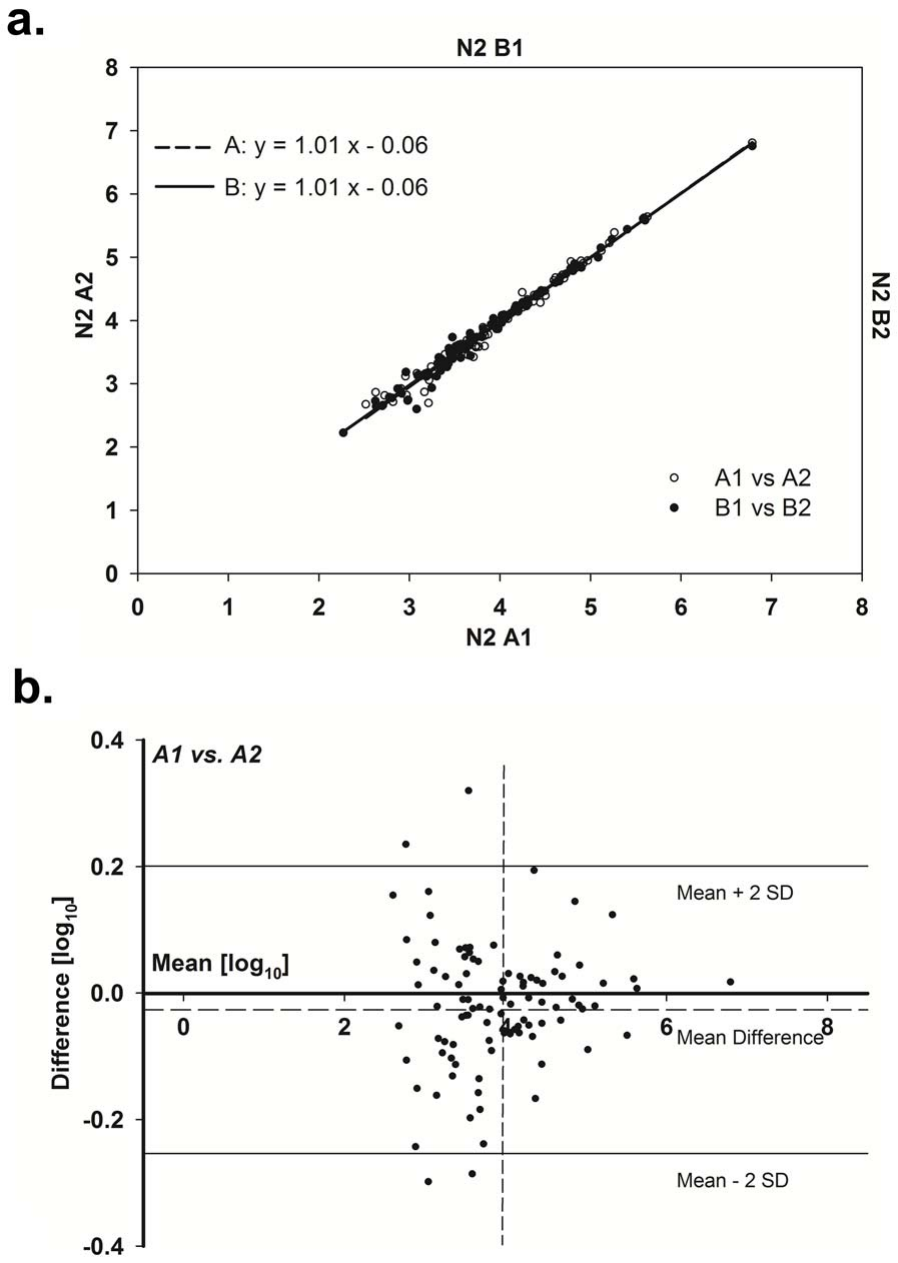
Assessment of technical and biological variability in samples. Replicate technical analyses were performed on each of two exometabolome samples collected from independent, normoxically-cultured, N2 worm preparations. For all four datasets, integrated metabolite peak areas were normalized to internal standard and total worm protein. Data were plotted after log_10_ transformation. (**a**) Combined scatterplot showing technical variation for both biological samples (A1 versus A2, B1 versus B2). (**b**) Bland-Altman plot highlighting differences between the integrated intensities of metabolites in A1 and A2. The bold horizontal line represents the average difference across all metabolites. The solid horizontal lines correspond to the mean difference ± two standard deviations. The dashed vertical line is the experimentally-determined noise threshold. Data below this threshold were removed from Figure 3 by applying a low-abundance cutoff.

To assess the degree of technical and sample variability within our procedures, two tests were undertaken. First, five replicate samples containing a set of 20 standard compounds (‘standard mix’) were individually derivatized and analyzed. With the exception of proline, coefficient of variation (CV) values for these technical replicates were in the range of 3–13%, and averaged 7.6% (Table 1). This level of variability is comparable to that of prior GC-MS metabolomics studies [14], [21], [22], [23]. Fiehn et al. [22], for example, analyzed 11 metabolites in *Arabidopsis thaliana* (n = 7) and found CV values ranging from 2–12%, averaging 5.8%. To assess sample variability between biological replicates, two independently-collected worm exometabolome samples were each split into equal aliquots and then all four samples were processed in parallel. Following normalization to the 3,4-dimethyoxybenzoic acid internal standard, and then to total protein, peak areas were log_10_ transformed and analyzed using scatterplots and Bland-Altman mean-difference plots (Figure 3). We observed excellent reproducibility in our sample sets, with the slopes of the lines-of-best fit through the scatterplots close to unity (Figure 3).

**Table 1:** Technical Reproducibility.

Metabolite	CV (%)
Pyruvate	6
Lactate	4
Alanine	6
Glycine	7
Valine	6
Leucine	7
Isoleucine	6
Proline	22
Succinate	7
Fumarate	5
Methionine	6
Serine	6
Threonine	3
2-Ketoglutarate	12
Phenylalanine	4
Malate	6
Aspartate	8
Glutamate	9
Citrate	13
Tyrosine	8

## Discussion

Analysis of the *C. elegans* exometabolome using GC-MS has provided novel insights into the metabolism of this species when exposed to anoxia. These results not only highlight the utility of using metabolomics-based methods for studies of worms, but also show conclusively that *C. elegans* is a mixed acid fermenter because multiple acidic end-products were detected in the exometabolome of anoxic worms, including lactic acid, succinic acid, 3-hydroxybutyric acid, 2-hydroxyglutaric acid and 2-hydroxybutyric acid. Elevated levels of lactic acid and succinic acid have been previously reported by Föll and colleagues [7]. Through generation of a five-point calibration curve, it was determined that the concentration of succinic acid in the exometabolome was 80±30 µM under normoxic conditions, and 1100±200 µM when animals were exposed to anoxia. Like its distant relative *Ascaris suum* [24], *C. elegans* also employs short-chain, unsaturated fatty acids (isovaleric acid, isobutyric acid, butyric acid, valeric acid and 2-methylbutyric acid) as terminal electron acceptors to remove reducing equivalents in the form of volatile waste products. The presence of these compounds in the exometabolome of anaerobically cultured worms confirms our previous postulation on their existence [9]. Cooper and Van Gundy previously determined that ethanol, acetaldehyde, and an unknown four-carbon alcohol were predominant anaerobic fermentative end-products of *Caenorhabditis* sp. [8] We were unable to confirm the production of ethanol or acetaldehyde using our methods since both compounds eluted in the GC solvent front and could not be quantified; based on the metabolites we did detect, it is possible that the four-carbon alcohol is 2-hydroxybutyrate. Our results indicate that the reductive state of *C. elegans* increases under anoxia. This is indirectly supported by accumulation of metabolites such as histidine, lysine, glutaric acid and xanthine (see below), all of which require NAD^+^ to be catabolized.

In prior studies, we hypothesized that *C. elegans* would make use of malate dismutation to increase the efficiency of ATP production during anoxic exposure [9]. By coupling mitochondrial complex I with rhodoquinone [25] and a fumarate reductase [26], ATP synthase can remain fully functional. Our detection of significantly decreased levels of all TCA intermediates except malate and succinate, the latter being almost 20 times higher in anaerobic worms, in addition to the appearance of multiple volatile fatty acids, several of which require rhodoquinone for their reductive biosynthesis (Figure 2), supports our contention that this pathway is active in anaerobic worms. The elevation of propionic acid under anaerobic conditions suggests that some succinic acid is metabolized further in anaerobic worms as an additional means of increasing ATP yield. Propionic acid was previously shown to be a major component of the *C. elegans* exometabolome when animals were exposed to anoxia at 20°C [7]. *C. elegans* also contains a functional glyoxylate cycle [27], [28]. This pathway is generally viewed as a means of allowing the products of fatty acid oxidation to be incorporated into glucose during embryonic development. Although we did not detect glyoxylic acid in the exometabolome of anaerobic worms, the observed increase in ethanolamine, valeric acid, glycine and succinate suggest that the glyoxylate cycle may also be active under these conditions (see Figure 2). Interestingly, the combination of malate dismutation, rhodoquinone and fumarate reductase would allow the glyoxylate cycle to act as a conduit for channeling acetyl CoA units directly into the mitochondrial ETC (by way of malate) rather than simply being a means for making glucose for glycolytic fermentation. If true, this would represent a previously unappreciated function for the glyoxylate cycle.

Several catabolic reactions involved in amino acid degradation require oxygen; these include reactions catalyzed by phenylalanine hydroxylase, prolyl-4-hydroxylase, tyrosinase, and tryptophan-2,3-dioxygenase (Figure 2). As expected, all of the substrates consumed by these reactions accumulated in anoxic worms, except tryptophan, which we have been unable to detect using our procedure. Tyrosinase, tryptophan-2,3-dioxygenase, and tryptophan decarboxylase are all involved in neurotransmitter production, and it is interesting that a common response to low oxygen of many species (including *C. elegans*) is to halt their physical movement. Xanthine was also observed to accumulate significantly in the exometabolome of anoxic worms (five fold), while uric acid significantly decreased (13 fold). The catabolism of xanthine to uric acid is catalyzed by xanthine dehydrogenase (XAD), an NAD^+^-requiring enzyme. Under certain conditions (including following limited proteolysis, cysteine oxidation, or alterations in osmolarity), XAD can be converted into xanthine oxidase, an oxygen-utilizing form of the enzyme. Why hypoxanthine, which is the precursor of xanthine and which is also a substrate for XAD, did not accumulate under anoxia while xanthine did, is unclear. It is possible that xanthine catabolism is rate limiting and hence as oxygen becomes limiting this product is the first to accumulate. This aside, our data show, unequivocally, that *C. elegans* utilizes uricogenesis for nitrogen waste excretion. Surprisingly, we also detected urea in the exometabolome of *C. elegans*. This observation can be explained by the fact that *C. elegans* has retained one enzyme of the urea cycle, namely arginase, and in *C. elegans*, arginine, instead of creatine, is used as a phosphagen [29]. One additional observation that we have made, and which we believe is very intriguing, involves the marked (34 fold) decline in sulfate production that occurred under anaerobia and which was accompanied by increased methionine excretion (10 fold). Recent studies have implicated a role for altered H_2_S levels in anoxia signaling [30]; the presence of three sulfide:quinone oxidoreductases in the *C. elegans* genome almost certainly indicates that this ancient signaling pathway is conserved in worms.

Finally, substantial amounts of the branched chain amino acids leucine, isoleucine and valine, were observed to accumulate in the exometabolome of anoxic worms. Using purified standards for absolute quantification, the following concentrations were recorded when comparing anaerobic worms to aerobic worms: valine, 1000±100 vs. 400±200 µM; leucine, 40±4 vs. 6±2 µM; and isoleucine, 40±4 vs. 5±2 µM. The aminotransferase that catalyzes the first step in the degradation of the branched-chain amino acids requires α-ketoglutarate, and in the exometabolome of anoxic worms this compound was markedly reduced (22 fold). The preferential accumulation of valine may reflect a difference in K_m_ for the aminotransferase. The catabolic reactions that follow deamination of the branched chain amino acids normally involve oxidative decarboxylation of the resulting α-ketoacid, its coupling to acetyl-CoA, and then oxidation of this intermediate in a reaction coupled to ubiquinone and the mitochondrial electron transport chain. For *A. suum* it is known that the redox potential of this latter reaction cannot supported by rhodoquinone [31]. This effectively provides a second block to forward flux through the branched chain amino acid catabolic pathways under anoxia.

Branched-chain amino acids have previously been shown by nuclear magnetic resonance (NMR) analysis to be markedly elevated in the tissues of long-lived *daf-2* and *daf-2;pept-1* mutants relative to wild-type (N2) animals [32], [33]. Metabolites present in spent culture medium were also shown to positively correlate with metabolites found in the internal milieu [32]. Our present findings raise a question regarding these findings. Specifically, to what extent does the accumulation of branched chain amino acids in *daf-2* and *daf-2;pept-1* mutants reflect their underlying metabolism or instead an enhanced susceptibility to an inadvertent anoxic challenge caused by the metabolite isolation procedure? In this regard, we note that Martin *et al.* [32] rotated 0.5 ml of packed animals in a volume of 9.5 mL for six hours in a 50-mL tube, opening the tube once during the procedure to allow air exchange. In our hands, similar-sized worm samples become anoxic in the space of a couple of minutes if not agitated vigorously and left continuously exposed to the atmosphere.

## Methods

### Materials

D-(+)-sucrose (99.7%) was obtained from Acros Organics (Cat. No. 177140050). Gelatin (Cat. No. G-2500), 3,4-dimethoxybenzoic acid (Cat. No. D131806, 99+%), 3-methylvaleric acid (Cat. No. 222453, 97%), anhydrous pyridine (Cat. No. 270970, 99.8%), methoxylamine HCl (Cat. No. 226904), agar (Cat. No. A7002), cholesterol (Cat. No. C8503, 95%), and acetonitrile (HPLC grade) were obtained from Sigma-Aldrich. Phenylpyruvic acid (Na salt, monohydrate, Cat. No. A14027, 98%) and L-norvaline (Cat. No. L08658, 99%) were obtained from Alfa Aesar. *N*-methyl-*N*-(*tert*-butyldimethylsilyl)trifluoroacetamide (MTBSTFA)/1% *tert*-butyldimethylchlorosilane (TBDMCS) was obtained from Thermo Scientific (Cat. No. TS-48927). Bacteriological peptone was obtained from Fluka (Cat. No. P0556).

### Worm Maintenance

*C. elegans* (N2 Bristol) were cultured on 10-cm BNGM agar plates coated with *E. coli* (OP50) lawns. Briefly, plates were prepared by autoclaving an aqueous suspension containing 1% w/v peptone, 2% w/v agar, and 50 mM NaCl. Following the autoclave cycle, calcium chloride (1 M), magnesium sulfate (1 M), potassium phosphate buffer (1 M, pH 6.0), and cholesterol (5 mg/mL in ethanol) were added to obtain final concentrations of 1 mM CaCl_2_, 1 mM MgSO_4_, 25 mM phosphate, and 5 µg/mL cholesterol. After allowing the plates to dry for two days, 500 µL of a 1∶750 dilution of OD_600_ 2.5 OP50 was spread onto each plate, and they were allowed to dry for an additional day before use. Plates not used immediately were stored at 4°C.

Cohorts of synchronously developing animals were established using a limited egg-lay strategy. Briefly, ∼30,000 one-day-old adults were allowed to lay ∼250,000 eggs onto three 10-cm BNGM/OP50 plates for four hours at 20°C. Eggs were collected into S-Basal (100 mM NaCl, 50 mM KH_2_PO_4_, pH 6.8), counted, plated at a concentration of 8,000 per 10-cm BNGM/OP50 agar plate, then allowed to mature at 20°C into one-day-old adults.

### Excreted Metabolite Collection

Collection of the *C. elegans* exometabolome was performed as described previously [5]. Briefly, 100,000 one-day-old adult worms were collected into 50 mL S-Basal, then bacteria and eggs removed by multiple washes (6×50 mL S-Basal, 1×g). Bacteria clinging to the cuticle, and dead or damaged animals, were removed by sucrose flotation [7] (10 mL 35% sucrose, 2,910×g, 3 min, 4°C). Worms on top of the sucrose cushion were transferred to a fresh tube and quickly washed with S-Basal (3×10 mL, 280×g, 20 sec). The first wash was performed at 4°C to minimize the response to residual sucrose. All subsequent washes were conducted at room temperature. (We have examined the exometabolomes of two different strains, both with and without sucrose flotation, and determined that this step does not result in overt alteration to the collected data Figure S2). All tubes and Pasteur pipettes were passivated prior to use by rinsing them with a solution of 0.5% gelatin, followed by deionized water, to reduce the number of animals sticking to the interior of glass- and plastic-ware. At the end of the last wash, the worms were weighed (wet pellet) and adjusted to a concentration of 600 mg of the wet pellet in a final volume of 1.4 mL S-Basal. Resuspended worms were then transferred to a 3.5-cm glass dish. The dish (without the lid) was placed in a hydration chamber and incubated at ambient conditions (22–23°C) with rotation at 100 rpm for 18 hr. In the case of anaerobic experiments, the dish was placed in a sealed container with a positive pressure of hydrated 500 ppm CO_2_ (balance nitrogen). After incubation the animals were collected and pelleted (400×g, 3 min, 22°C). The supernatant was filtered through a 0.2-µm nylon filter (Life Science Products, Inc., Frederick, CO; Cat. No. 6502-413X) then the filtrate and pellet stored separately at −80°C until analysis.

### Sample Derivatization

Plasticware from which plasticizers were confirmed not to leak, were employed for the following reactions. In a 2-mL polypropylene tube, (Eppendorf; Cat. No. 022363352), the following was combined: 100 µL sample (N2 exometabolome, S-Basal blank, or a mix of standards – see following paragraph), 10 µL each of 10 mM 3,4-dimethoxybenzoic acid (in water, made basic with NaOH to pH∼10), 5 mM L-norvaline, and 5 mM phenylpyruvic acid (these three compounds served as internal standards), and 575 µL acetonitrile (to form an azeotrope with water). The blank permitted identification of derivatization artifacts. Selected standard compounds (representing a variety of chemical and functional groups, including carboxylic acids, keto acids, and amino acids) were also derivatized for quality control. The samples were dried by vacuum centrifugation overnight (approximately 15 hr) without heating. Samples were then derivatized *in situ* using a modified version of the derivatization procedure of Paik and colleagues [34], [35]. To dried samples, 40 µL of methoxylamine HCl (20 mg/mL in dry pyridine) was added. The samples were topped with dry nitrogen and incubated at 30°C for 90 min with periodic mixing. Following methoximation, 60 µl of derivatization reagent (MTBSTFA/1% TBDMCS) was added and the samples were again topped with dry nitrogen and allowed to incubate at 80°C for 1 hr with periodic mixing. Derivatized samples were centrifuged for 3 min at 2,000× G, and 50 µL of each sample was transferred to glass inserts inside autosampler vials. Vials were topped with dry nitrogen, sealed with Teflon-lined caps, and analyzed within 48 hr.

The mix of standards was prepared by adding 30 µL of a solution containing 1-mM of each of the following to 100 µL S-Basal: citramalic acid, citric acid, ethanolamine, fumaric acid, 2-hydroxybutyric acid, 3-hydroxy-3-methylbutyric acid, hypoxanthine, α-ketoglutaric acid, lactic acid, malic acid, 3-methyl-2-oxobutanoic acid, 3-methyl-2-oxopentanoic acid, 4-methyl-2-oxovaleric acid, 2-oxobutyric acid, pyruvic acid, succinic acid, urea, and xanthine. Five microliters of Amino Acid Standard H (Pierce, Cat. No. 20088) containing alanine, alanine, arginine, aspartic acid, cystine, glutamic acid, glycine, histidine, isoleucine, leucine, lysine, methionine, phenylalanine, proline, serine, threonine, tyrosine, valine, each at 2.5 mM, and cysteine at 1.25 mM, was then added.

To analyze volatile (C3–C6) metabolites which would otherwise be lost in the vacuum drying step described above, we modified the procedure of Morrison et al. [36] To 100 µL of sample, 10 µL of 10 mM 3-methylvaleric acid was added as an internal standard, and the solution was then acidified by addition of 20 µL of 1 M HCl. Subsequently, metabolites were extracted by addition of 300 µL diethyl ether and vortexing at room temperature for 30 min. The organic (upper) layer was removed and concentrated to approximately 50 µL under a gentle stream of nitrogen. Methoximation was performed by addition of 40 µL of 20 mg/mL methoxylamine HCl in pyridine followed by incubation for 90 min at 30°C, as described above. Generation of *t*-BDMS derivatives was then accomplished by addition of 30 µL of MTBSTFA/1% TBDMCS and incubation at 80°C for 30 min. Samples were transferred to autosampler vials and analyzed by GC-MS within 24 hr.

### GC-MS Analysis

Gas chromatography-mass spectrometry (GC-MS) analyses were performed on a Thermo Fisher (San Jose, CA) TRACE DSQ single quadrupole mass spectrometer. GC conditions for standard exometabolome samples were as follows: column, ZB-5MS (Phenomenex; Torrance CA), 30 m×0.25 mm, 0.25 µm film thickness; carrier gas, helium; linear velocity, 1 mL/min (constant flow); injection, split, 10 mL/min split flow; injector temperature, 220°C; column temperature program, initial temperature of 70°C held for 1 min followed by an increase to 310°C at 5°C/min. MS conditions were: ionization, electron impact (70 eV); detection, positive ion; full scan analyses, *m/z* 50–*m/z* 700 at two scans/sec. Volatile metabolites eluted with the solvent front using this method, so GC separation of these analytes started with an initial temperature of 50°C held for 1 min, followed by an increase to 80°C at 10°C/min. The temperature was maintained for 3 min at 80°C after which it was increased to 275°C at a rate of 30°C/min.

### Total Protein Determination

Protein was extracted from frozen worm pellets by boiling in 1% sodium dodecyl sulfate (10 min, final volume, 5 mL). Samples were vortexed halfway through the extraction procedure. Insoluble cuticle shells were separated from solubilized protein by centrifugation (17,000×g, 5 min). The remaining soluble fraction was referred to as “total protein.” The bicinchoninic acid assay (Pierce, Rockford, IL; Cat. No. 23225), using bovine serum albumin as standard, was used to determine protein concentration. For most samples, a 1∶20 dilution was sufficient to obtain A_562_ readings in the linear portion of the BSA standard curve. Concentrations were determined either immediately after boiling, or if samples were re-frozen, they were reheated for 10 min in boiling water and centrifuged again prior to analysis.

### Peak Deconvolution and Integration

The mass spectral library used in this study was derived, in part, from a freely available *t*-BDMS-derivative library provided by the Max Planck Institute in Golm, Germany. The library was truncated to include only compounds found in our *C. elegans* exometabolome samples [16]. Total ion count traces were deconvoluted using using *AMDIS (v. 2.1)*. Unidentifiable mass spectra were given a unique code and added to our library. This process created a custom database that was specific for our samples. We attempted to identify any peaks which were differential between the two experimental groups, but were not initially assigned to a metabolite, through matching to other libraries, the literature, or by interpretation through visual inspection. When these assignments were confirmed against purified standards the library was updated accordingly. *MET-IDEA (v. 2.0)* [37] was used for peak integration. Typical chromatography parameters used for integration with *MET-IDEA* were - average peak width: 0.15, minimum peak width: 0.5× average peak width, maximum peak width: 3× average peak width, and peak start/stop slope: 1.5. MS parameters used were - mass accuracy: 0.1, and mass range: +/−0.2.

### Preprocessing the Peak List

Preprocessing consisted of removing artifact peaks, normalization to the internal standard (3,4-dimethoxybenzoic acid), and normalization to total protein. Peaks that were present in blank samples consisting of S-Basal were considered to be derivatization artifacts and were removed from the *MET-IDEA* peak list. This process adjusted for sample loss, variation in instrument sensitivity and the total protein mass of animals used in each experiment.

### Hierarchical Clustering

Clustering was performed using the HierarchicalClustering module of the GenePattern software suite [38] (http://www.broad.mit.edu/cancer/software/genepattern/). Metabolites were clustered using the pairwise complete-linkage method. Distance measures on the clustered data were calculated using a Pearson Correlation.

### Quality Control

Technical replicates of the standards mix were analyzed, and coefficients of variation (CV) were calculated. Technical replicates of biological exometabolome samples were analyzed by scatterplots and mean-difference (Bland-Altman) [39] plots after transforming to a log scale and plotting in *Sigmaplot v. 11.0* (Systat Software, Inc.).

### Statistics

The filtered and corrected peak intensities for each metabolite approximated a log-normal distribution when compared across samples, therefore data were log-transformed prior to analysis. Normoxic and anoxic samples were each collected on two different dates in multiple independent experiments, therefore, a randomized-block model was used to evaluate the effect of anoxia, with date of collection as the random effect variable and oxygen status as the main effect of interest. For each exometabolite, the null hypothesis that the oxygen effect was not significantly different from zero was tested by calculating a t-statistic from the fitted coefficient and its standard error, and comparing it to a t-distribution. This is equivalent to a two sample t-test except that any batch effects due to which date a sample was collected were accounted for by the random effect variable; the degrees of freedom were accordingly reduced by one for the two distinct collection dates. To correct for multiple comparisons, the false discovery rate (FDR) was constrained at 5% using the method of Benjamini and Hochberg, [39]; which, for the non-volatile metabolites corresponded to a significance threshold of p≤0.03377, and for the volatile metabolites, p≤0.00988. The R statistical language [40] was used for the FDR calculation and the nlme [40], [41] R package was used for the randomized-block model and hypothesis tests. For plotting purposes normalized data that was further corrected for collection date, if the random-effect variable was significant, was employed (all raw and adjusted data is provided in the supporting information File S1).

### Metabolic Mapping

Metabolic maps were prepared using VANTED (v. 2.0). This software is freely available and can be downloaded from http://vanted.ipk-gatersleben.de/. Metabolic connections were determined using the KEGG Mapper function provided by the Kyoto Encyclopedia of Genes and Genomes (KEGG), which can be found at http://www.genome.jp/kegg/mapper.html.

## Supporting Information

Figure S1 **Response curves for GC-MS analysis of selected metabolites.** Four-point GC-MS response curves for alanine, isoleucine, leucine, succinate, and valine were established by integrating the peak at each analyte's established retention time in an extracted ion chromatogram corresponding to a characteristic fragment for that compound (usually [M-57]^+^). The integrated peak area of the extracted ion chromatogram was normalized relative to the internal standard (3,4-dimethoxybenzoic acid), then a scaling factor was applied (For presentation purposes the ordinate values have been multiplied by 10^4^). The slope of the least-squares plot between quantification ion intensity and analyte concentration is metabolite-specific - reflecting differences in derivatization efficiency, differences in ionization efficiency, and/or our choice of ion (*m/z*) used for quantification.
(TIF)

Figure S2 **Effect of sucrose wash step on exometabolome composition.** 250,000 one day old adult N2 and MQ1333 [*nuo-6(qm200)*] worms were prepared for exometabolome collection exactly as described under Materials & Methods. Each worm sample was split into two equal fractions and processed identically, except only one fraction from each pair was subjected to sucrose flotation. Following an 18 hr collection period under ambient oxygen conditions, excreted metabolites were collected and analyzed by GC-MS and hierarchical clustering (based on Pearson's correlation coefficient). A low-abundance cut-off filter was applied. Heat map is colored according to global variation among metabolites over the entire exometabolome data set (blue-red).
(TIF)

File S1 **Raw data and statistical analyses.**
(XLS)

File S2 **Rea Lab mass spectral library.**
(MSP)
