# Supplementary figures and images for: Profiling the Anaerobic Response of C. elegans Using GC-MS
Source: PLoS One. 2012 Sep 27;7(9):e46140. doi: 10.1371/journal.pone.0046140 (PMC3459875; doi:10.1371/journal.pone.0046140)

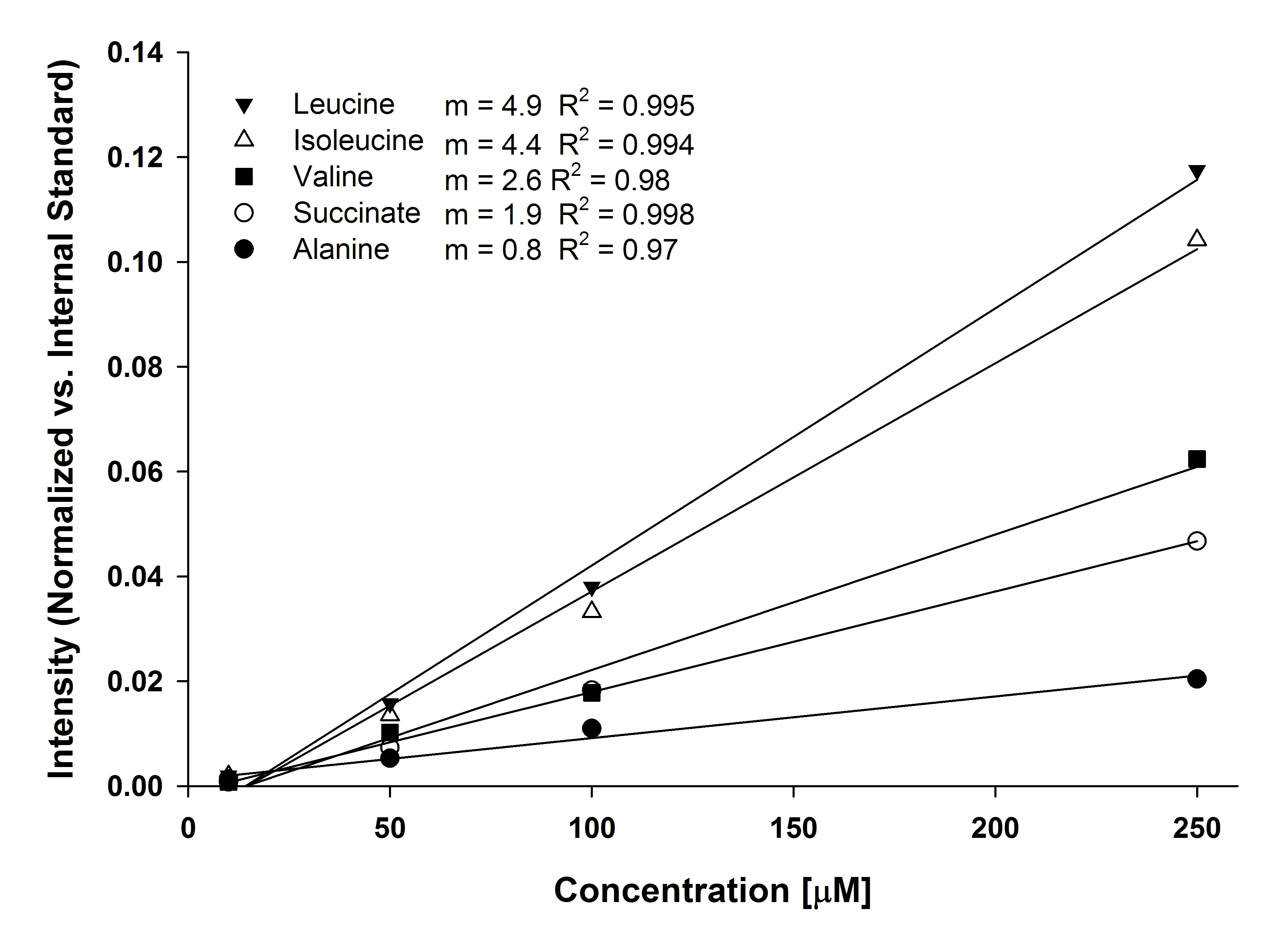

Supplement: Figure S1 — Response curves for GC-MS analysis of selected metabolites. Four-point GC-MS response curves for alanine, isoleucine, leucine, succinate, and valine were established by integrating the peak at each analyte's established retention time in an extracted ion chromatogram corresponding to a characteristic fragment for that compound (usually [M-57]+). The integrated peak area of the extracted ion chromatogram was normalized relative to the internal standard (3,4-dimethoxybenzoic acid), then a scaling factor was applied (For presentation purposes the ordinate values have been multiplied by 104). The slope of the least-squares plot between quantification ion intensity and analyte concentration is metabolite-specific - reflecting differences in derivatization efficiency, differences in ionization efficiency, and/or our choice of ion (m/z) used for quantification. (TIF) [file pone.0046140.s001.tif]

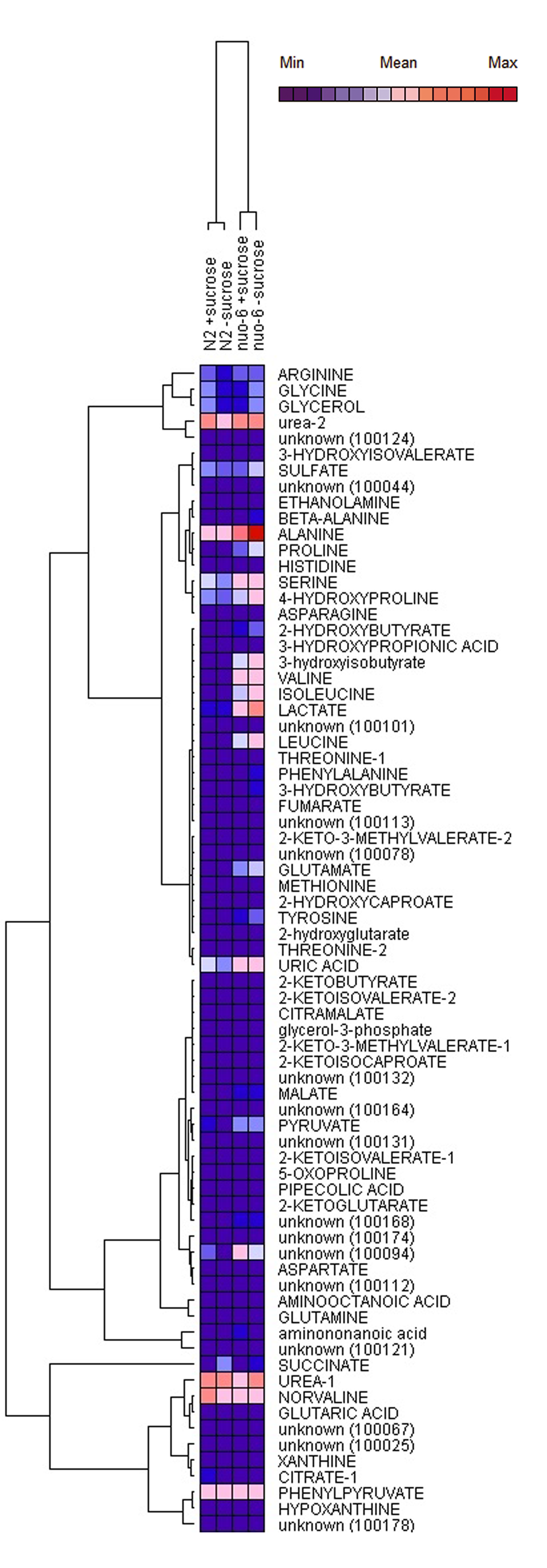

Supplement: Figure S2 — Effect of sucrose wash step on exometabolome composition. 250,000 one day old adult N2 and MQ1333 [nuo-6(qm200)] worms were prepared for exometabolome collection exactly as described under Materials & Methods. Each worm sample was split into two equal fractions and processed identically, except only one fraction from each pair was subjected to sucrose flotation. Following an 18 hr collection period under ambient oxygen conditions, excreted metabolites were collected and analyzed by GC-MS and hierarchical clustering (based on Pearson's correlation coefficient). A low-abundance cut-off filter was applied. Heat map is colored according to global variation among metabolites over the entire exometabolome data set (blue-red). (TIF) [file pone.0046140.s002.tif]
